# The role of mental health in primary prevention of sexual and gender-based violence

**DOI:** 10.3402/gha.v7.24741

**Published:** 2014-09-12

**Authors:** Aník Gevers, Elizabeth Dartnall

**Affiliations:** 1 Gender and Health Research Unit, South African Medical Research Council, Tygerberg South Africa; 2 Department of Psychiatry and Mental Health, University of Cape Town, Cape Town, South Africa; 3 Sexual Violence Research Initiative, Pretoria, South Africa

**Keywords:** primary prevention, mental health, sexual violence, gender-based violence, intervention

## Abstract

In this short communication, we assert that mental health has a crucial role in the primary prevention of sexual and gender-based violence (SGBV). However, we found that most research and practice to date has focused on the role of mental health post-violence, and SGBV primary prevention is relying on public health models that do not explicitly include mental health. Yet, key concepts, processes, and competencies in the mental health field appear essential to successful SGBV primary prevention. For example, empathy, self-esteem, compassion, emotional regulation and resilience, stress management, relationship building, and challenging problematic social norms are crucial. Furthermore, competencies such as rapport building, group processing, emotional nurturing, modelling, and the prevention of vicarious trauma among staff are important for the successful implementation of SGBV primary prevention programmes. SGBV primary prevention work would benefit from increased collaboration with mental health professionals and integration of key mental health concepts, processes, and skills in SGBV research.

Sexual and gender-based violence (SGBV) can have a profound and life-long impact on the mental health of survivors who are at increased risk of depression, anxiety, and post-traumatic stress disorder (1–3). Children exposed to violence and abuse are at risk of experiencing or perpetrating violence as an adult (4–6). The important role mental health interventions play in mitigating the mental health impacts of SGBV is well recognised in the research literature (7–11). Interventions include counselling or therapeutic or rehabilitative services for survivors, perpetrators, and affected family members, and have been shown to reduce negative psychological sequelae of rape and risk of revictimisation (6, 12–14).

Much of the mental health intervention research has investigated the effectiveness of these interventions in treating PTSD (12–14) although it is not clear from the literature whether any of these interventions is more effective than another or the extent to which these interventions have sustainable long-term impacts. Research on the integration of mental health into primary care is growing (15–18) and these innovations should include attention to providing mental health services for SGBV survivors which is particularly challenging in resource-poor settings (19). Some studies have found using lay people to deliver CBT to SGBV survivors in extremely fragile settings to be effective in alleviating mental health symptoms (20–22).

Increasingly, researchers are recognising the intergenerational cycle of violence (23, 24) and the linkages between trauma exposures in childhood and increased risk of psychological ill-health, and experiencing violence and perpetrating violence later on in life (4–6). Work with children and parents is also encouraging in terms of preventing and reducing the emotional effects of trauma (25–30). There is however a paucity of published research clarifying the role of mental health in the primary prevention of SGBV. Indeed, a search for such articles yielded a focus on violence prevention as a strategy to prevent mental health disorders (25, 30).

The field of SGBV primary prevention is relatively young and has been advanced by recent research investigating the factors contributing to the perpetration of such violence (31–36). These large-scale surveys have informed a public health approach to the primary prevention of SGBV (37). Current models driving SGBV primary prevention approaches promote ‘(a) building gender equality and challenging hegemonic masculinities; (b) challenging the widespread acceptance of violence; (c) improving conflict resolution and communication skills; (d) developing relationship-building skills; (e) reducing substance abuse; and (f) improved gun control’ (38, p. 14). Though the role of mental health is often not explicit in SGBV primary prevention models, it is an essential component in successful interventions in this burgeoning and crucial field with a role in various aspects of SGBV primary prevention, including strengthening protective factors within individuals, strengthening protective factors within relationships and environments, and strengthening the capacity of service providers to deliver interventions.

## Understanding and strengthening protective factors within individuals

Violence prevention interventions should address the mental health, coping skills, and social skills of intervention participants. Empathy and compassion, for example, have been identified as integral to violence prevention at an individual level; that is, if individuals understand the level of harm that violence causes others and accept their responsibility not to harm others, individuals will be less likely to use violence (39). Similarly, research with men in prison highlights the importance of promoting strong self-esteem and social skills to help individuals withstand perceived dishonour, disrespect, shame, rejection, humiliation, or insult by other individuals (39, 40). Formative research in the early stages of intervention development can help to identity the areas of mental health that are relevant within a local context. For example, formative research done with teenagers and caregivers to inform the development of multi-faceted school-based SGBV primary prevention intervention drew attention to the need to include modules on stress (as a popular concept encompassing typical depression, anxiety, and psychosomatic symptoms as well as feeling overwhelmed, tense, irritable, and short-tempered), coping and emotional regulation in addition to the components identified within the public health models described earlier. Both caregivers and teenagers identified mental health issues as particular challenges in their lives which they felt exacerbated violence and strained relationships in their homes. Part of the stress and coping strategies should include teaching individuals to tolerate distressing emotions and express them respectfully instead of externalising them which increases the risk of violence. Violence prevention interventions will be strengthened with the integration of tools to build the mental health, well-being, and emotional resilience of participants.

## Strengthening protective factors within relationships and environments

Gender attitudes, childhood exposures to violence, and growing up in toxic environments are key factors underlying SGBV (34, 40). Nurturing and emotionally supportive relationships during childhood are likely protective factors against later SGBV perpetration and victimisation. Indeed, secure attachment and a sense of connectedness are core human needs (41, 42) and facilitate the development of a stronger self-esteem, empathy, and emotional regulation. Poor parenting and insecure attachment have been shown to predict later behavioural problems and delinquency (43, 44). Further, these behavioural problems persist and can worsen if the child is living in high-risk social contexts (45, 46). Promoting supportive and nurturing family relationships or relationships with other positive individuals or groups would support a primary prevention agenda. The mental health field can also contribute to understanding and harnessing relationship dynamics within couples to support primary prevention; that is, respectful and caring relationships between parents will contribute to positive role modelling of constructive, open communication styles, negotiation skills, and compromise for young people. Indeed, should these relationship dynamics become normative they would significantly contribute to the primary prevention of SGBV.

Changing social norms that support SGBV is a key aspect of violence prevention (38). Many social norms are helpful and promote pro-social behaviour, but in some settings, prevailing social norms enable men to perpetrate violence against women and children with impunity (38). The social psychology literature on social norms, and their influence on group behaviour, social conformity, and pro-social behaviour can help inform theoretical frameworks for primary prevention interventions.

The research on learned helplessness, resilience, and empowerment may contribute to strategies to challenge social norms of gender inequality. Such discrimination can often have an adverse impact on mental health (47), and the pervasiveness of such discrimination and the stress of trying to conform to or achieve impossible gender ideals may lead to a sense of learned helplessness. Such a reaction may promote passivity and acceptance of social norms. However, a positive feedback loop may be driven by resilience and empowerment types of responses and these may contribute to changing gender inequality and social norms.

## Strengthening the capacity of intervention facilitators and service providers to deliver SGBV interventions

Intervention facilitators must guide participants through a process of change. To do this successfully, they need rapport-building skills, behavioural observation skills, and group processing skills. These interpersonal skills are core competencies of mental health service providers indicating another key point of integration between the SGBV primary prevention and mental health fields. Facilitators must have the skills to model the types of relationships and interaction patterns the SGBV primary prevention intervention promotes. SGBV primary prevention programmes address very personal and sensitive beliefs, attitudes, and behaviours. Given the sensitive nature of programme content, the intervention sessions may elicit mental health distress or resistance that the facilitators need to deal with and resolve in order to continue with the intervention. Thus, management of these mental health issues within the group and group processes are essential skills for the successful delivery of SGBV interventions. It is essential that training and on-going support and supervision of facilitators assess and build these skills.

The mental health of staff working on SGBV primary prevention interventions is also hugely important. As noted, these interventions often confront very sensitive and very difficult concepts and skills which may precipitate emotional distress, cognitive dissonance, defensiveness or disclosure of past traumas and managing these reactions and disclosures can take a heavy toll on facilitators’ mental health and well-being. Indeed, vicarious trauma has been recognised as a significant risk to people working in the SGBV field; mental health issues that may arise in the wake of vicarious trauma include compassion fatigue, depression, anxiety or PTSD-type symptoms, and burnout (48). Staff safety and strategies for the prevention and responses to vicarious trauma should be considered when working on SGBV primary prevention projects (48).

## Development of research tools and methods

Integration of mental health in SGBV primary prevention is hampered by a paucity of validated, simplified mental health scales and tools. Tools developed in the global north are not necessarily valid in other settings, nor can the methods used to measure various psychological outcomes be easily applied in community settings or self-complete surveys. Researchers in SGBV and mental health fields need to work together to develop mental health measures and valid tools to measure them. This measurement would facilitate the integration of mental health skills and concepts into the theoretical models of SGBV primary prevention that drive the field.

## Conclusions

In summary, we argue that mental health interventions can and should be incorporated into SGBV primary prevention efforts at every level including creating change at the individual, interpersonal, and community levels as well as supporting those who are conducting and implementing this work. We highlight how mental health processes complement and aid existing models for SGBV primary prevention. Key concepts include increased empathy, compassion, self-esteem, and emotional regulation or coping. The mental health of participants and staff is also crucial for successful SGBV primary prevention. The development and integration of appropriately validated and standardised tools and measures for mental health into SGBV primary prevention research is essential in order to strengthen collaborations across these fields. We recommend the development of a joint research agenda on the role of mental health in primary prevention of SGBV and the creation of a joint learning initiative for mental health and SGBV prevention practitioners, advocates, and researchers.
